# Pulmonary hyalinizing granulomas

**DOI:** 10.36416/1806-3756/e20240382

**Published:** 2025-07-22

**Authors:** Edson Marchiori, Bruno Hochhegger, Gláucia Zanetti

**Affiliations:** 1. Universidade Federal do Rio de Janeiro, Rio de Janeiro (RJ) Brasil.; 2. University of Florida, Gainesville (FL) USA.

**Figure 1:**
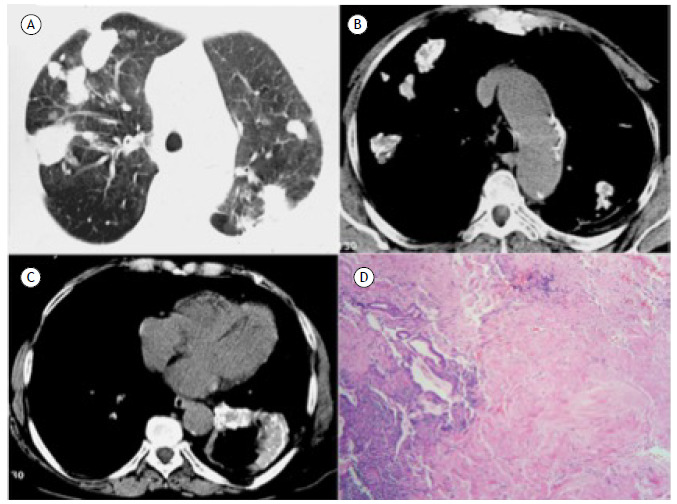
Chest CT images obtained with lung (A) and mediastinal (B and C) window setting showing multiple nodules of various sizes and irregular contours in both lungs, with soft-tissue density and foci of calcification within them. A cavitated mass with thick, irregular, partially calcified walls is also visible in the left lower lobe (C). In D, histological staining confirmed the deposition of hyaline tissue masses composed of hypocellular collagen lamellae, accompanied by sparse lymphocytic infiltrate that compresses and distorts the remaining bronchioles (H&E; original magnification, ×40).

A 28-year-old female patient presented with dyspnea, cough, chest pain, and weight loss. She reported having had tuberculosis 20 years prior. Chest CT) showed multiple, partially calcified pulmonary nodules and a cavitated mass (Figures 1A-C). Open lung biopsy revealed inflammatory lesions consisting of mature fibrous tissue, with areas of calcification and ossification infiltrated by small numbers of lymphocytes and plasma cells, consistent with hyalinizing granulomas (Figure 1D). Pulmonary hyalinizing granuloma (PHG) is a rare benign lung disease characterized by fibrosing nodules consisting of central whorled deposits of lamellar-collagen hyaline. The probable etiology of this condition is an exaggerated immune response to the antigenic stimuli of infectious agents, such as tuberculous bacilli and histoplasma organisms, or an autoimmune process. Because the symptoms are absent or mild, most PHG lesions are found incidentally by radiological examination and are not initially diagnosed correctly. Chest radiography and CT show solitary or multiple randomly distributed nodules and/or masses with or without calcification. Although rare, cavitation has been reported. The prognosis is generally excellent, although some patients develop retroperitoneal fibrosis or sclerosing mediastinitis.^1^ ^-^ ^4^ PHG should be considered in the differential diagnosis of pulmonary nodules or masses, even those that are cavitary or contain calcifications.
